# Liver Lipodystrophy in Barraquer–Simons Syndrome: How Much Should We Worry About?

**DOI:** 10.3390/life16010156

**Published:** 2026-01-17

**Authors:** Doina Georgescu, Daniel Florin Lighezan, Roxana Buzas, Paul Gabriel Ciubotaru, Oana Elena Țunea, Ioana Suceava, Teodora Anca Albu, Aura Jurescu, Mihai Ioniță, Daniela Reisz

**Affiliations:** 1 Department of Internal Medicine I, Center of Advanced Research in Cardiovascular Diseases and Hemostaseology, University of Medicine and Pharmacy Victor Babes, 2 Eftimie Murgu Plaza, 300041 Timisoara, Romania; doina.georgescu@umft.ro (D.G.); ancusa.oana@umft.ro (O.E.Ț.);; 2 Department of Physics, West University of Timisoara, 4 Vasile Parvan Blvd, 300223 Timisoara, Romania; 3 Department of Morphopathology, Microscopic Morphology, University of Medicine and Pharmacy Victor Babes, 2 Eftimie Murgu Plaza, 300041 Timisoara, Romania; jurescu.aura@umft.ro; 4 Department of Neurosciences, University of Medicine and Pharmacy Victor Babes, 2 Eftimie Murgu Plaza, 300041 Timisoara, Romania; reisz.daniela@umft.ro

**Keywords:** partial acquired lipodystrophy, liver lipodystrophy, MASH

## Abstract

Lipodystrophy is a rare group of metabolic disorders characterized by the abnormal distribution of body fat, which can lead to various metabolic complications due to the body’s inability to adequately process carbohydrates and fat. We report the case of a female, aged 53 years, who was admitted as an outpatient for progressive weight loss of the upper part of the body (face, neck, arms, and chest), dyspeptic complaints, fatigue, mild insomnia, and anxious behavior. Her medical history was characterized by the presence of dyslipidemia, hypertension, and a minor stroke episode. However, she denied any family-relevant medical history. Although the clinical perspective suggested a possible late onset of partial acquired lipodystrophy, due to the imaging exam that revealed an enlarged liver with inhomogeneous structure with multiple nodular lesions, scattered over both lobes, a lot of lab work-ups and complementary studies were performed. Eventually, a liver biopsy was performed by a laparoscopic approach during cholecystectomy, the histology consistent with metabolic disease-associated steatohepatitis (MASH). In conclusion, given their heterogeneity and rarity, lipodystrophies may be either overlooked or misdiagnosed for other entities. Barraquer–Simons syndrome (BSS) may be associated with liver disease, including cirrhosis and liver failure. Liver lipodystrophy in BSS may sometimes feature steatosis with a focal, multi-nodular aspect, multiplying the diagnostic burden. Liver lipodystrophy may manifest as asymptomatic fat accumulation but may progress to severe conditions, representing one of the major causes of mortality in BSS, apart from the cardio-vascular comorbidities. Given the potential of severe outcomes, it is mandatory to correctly assess the stage of liver disease since the first diagnosis.

## 1. Introduction

Lipodystrophy is a rare group of metabolic disorders characterized by abnormal distribution of body fat, which can lead to various metabolic complications due to the body’s inability to adequately process carbohydrates and fat. They are either inherited or acquired and can manifest as generalized or partial lipodystrophy. Partial lipodystrophy syndromes are a heterogeneous group of diseases characterized by selective loss of body fat that can be inherited (familial partial lipodystrophy) or acquired (acquired partial lipodystrophy). Limited and dysfunctional peripheral fat depots, along with relatively low levels of adipokines (such as leptin and adiponectin) in partial lipodystrophy, result in ectopic accumulation of fat and trigger the development of severe insulin resistance [1].

Partial lipodystrophy is considered a multisystem disease, associated with deficiency or dysfunction of adipose tissue, that may result in multiple cardio-metabolic issues with insulin resistance, diabetes, and dyslipidemia.

Barraquer–Simons syndrome (BSS) represents a rare form of acquired partial lipodystrophy (APL) of unknown etiology, with progressive evolution characterized by gradual and symmetrical loss of fat in the upper part of the body. Besides the metabolic issues that characterize all lipodystrophy conditions, this syndrome may be associated with various immune issues, hypocomplementemia, and renal disturbances [2].

Despite the tremendous achievements related to work-up advances, the clinical history and physical examination still represent the most important assets in recognition of a lipodystrophy disorder, by revealing a distinct body shape associated with particularities of the body composition and metabolic disturbances [3,4].

Liver lipodystrophy features entities associated with hepatocytes lipid deposition in patients with various forms of lipodystrophy, secondary to abnormal fat metabolism and its body distribution. The liver is frequently targeted by this metabolic condition with consecutive fatty accumulation. This can lead to so-called metabolic dysfunction-associated steatotic liver disease (MASLD), metabolic disease-associated steatohepatitis (MASH), characterized by inflammation and hepatocellular death, or even more severe hepatic pathologies like end-stage chronic liver disease, liver fibrosis, cirrhosis, and its complications, including hepatocellular carcinoma [5,6,7,8].

Liver lipodystrophy, where the hallmark is the hepatic fat build-up, is basically diagnosed by imaging studies and, in selected cases, confirmed by the anatomopathological examination. Hepatic steatosis may manifest as diffuse fat accumulation in the majority of cases or, in rare cases, as parcellar or focal steatosis [9,10,11].

MASLD, as a fatty liver feature, is shared by both entities: the lipodystrophy and the common metabolic syndrome. The most important difference between MASLD secondary to metabolic syndrome and MASLD from liver lipodystrophy is represented by the decrease in the range of the hormones produced by the adipocytes. Leptin and adiponectin represent the most important adipocyte-derived hormones with a key role in appetite control, insulin sensitivity regulation, and hepatocytes’ physiology.

Some authors consider that liver lipodystrophy should be taken into consideration when liver steatosis is found in lean patients with no history of alcohol abuse or exposure to toxins.

Recent studies reported a good positive correlation between the triglyceride range and the severity of liver steatosis, highlighting once more the impact of visceral adiposity on the fatty liver development in patients with partial lipodystrophy [12].

Given the early manifestation of the fatty liver disease and the high risk for fibrosis development in patients with partial lipodystrophy, it is mandatory to correctly assess the severity of fibrosis since the first diagnosis of the liver disease. Work-ups like FIB-4, Fibromax, combined with noninvasive imaging studies like hepatic ultrasonography, controlled attenuation parameter, and liver elastography by shear wave method or by transient elastography, should be the first to perform when it comes to assessing MASH in patients with partial lipodystrophy. In selected cases, MRI elastography or pathologic examination may be required in order to exclude advanced liver disease [13,14,15].

Informed consent was obtained from the subject involved in this study. This study was conducted in accordance with the Declaration of Helsinki and approved by the Institutional Ethics Committee of Emergency Clinical County Hospital, “Pius Brânzeu”, from Timisoara, Romania, protocol code 556/24 July 2025.

## 2. Materials and Methods

### Detailed Case Description

A female patient, aged 53, was admitted as an outpatient for dyspeptic complaints, fatigue, and progressive weight loss in the upper part of the body. She was in menopause for six years and had a long history of smoking cigarettes. Her narrative also included the story of an insidious weight loss over the last few years, with no obvious cause. She was also known for hypertension, mixed dyslipidemia, a recently transient cerebral ischemic attack and mild anxiety/depression disorder. However, she denied any family-relevant medical history.

Physical examination revealed a female patient in her early fifties, with symmetric fat loss of the face, the disappearance of Bichat’s fat bubble, which gave her a much older appearance, a lean aspect of the neck and shoulders, and thin arms and forearms. Her BMI was 21 kg/m^2^. Skin examination did not reveal acanthosis nigricans or signs of dermatomyositis. Skin fold thickness value assessed at the anterior arms was about 5 mm, and at the anterior thigh was about 20 mm. Two soft subcutaneous formations, one at the posterior cervical zone and the other at the anterior, median aspect of the inferior sternal zone, were observed, highly suggestive of lipomas. Body measurements such as neck circumference = 32 cm, waist circumference = 88 cm, and thigh circumference = 64 cm were also performed. APL is characterized by fat loss that spreads through a cephalocaudal distribution from the face, neck, shoulders, arms, and forearms and that extends to the thoracic region and upper abdomen. She had a normal respiratory frequency and no rales. Cardio-vascular examination revealed that BP = 130/70 mmHg, under hypotensive drugs, the cardiac rate = 68 beats/min, normal cardiac sounds, with no other pathological aspects. Her abdomen was slightly distended with mild tenderness at the level of the right upper quadrant, and the liver was enlarged, being accessible for palpation at 3 cm under the right costal rib line. The patient had normal bowel movements, normal diuresis, and no neurological signs.

**Figure 1:**
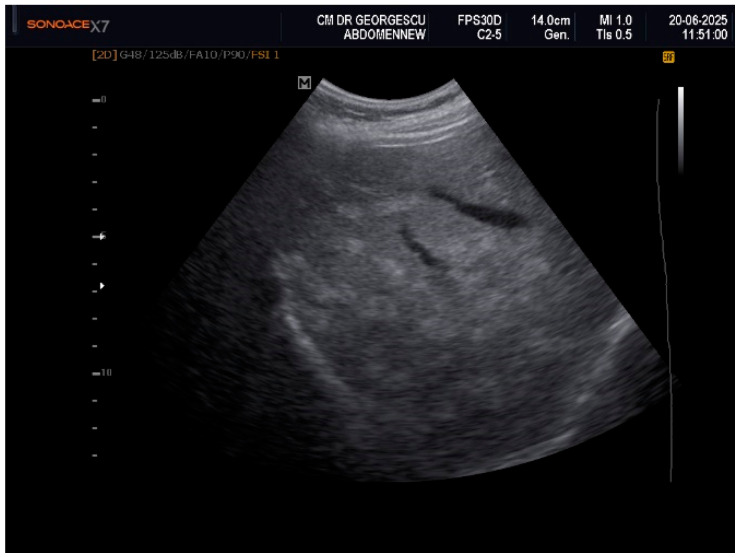
Two-dimensional ultrasound exam features of scattered hyperechoic nodules.

**Figure 2:**
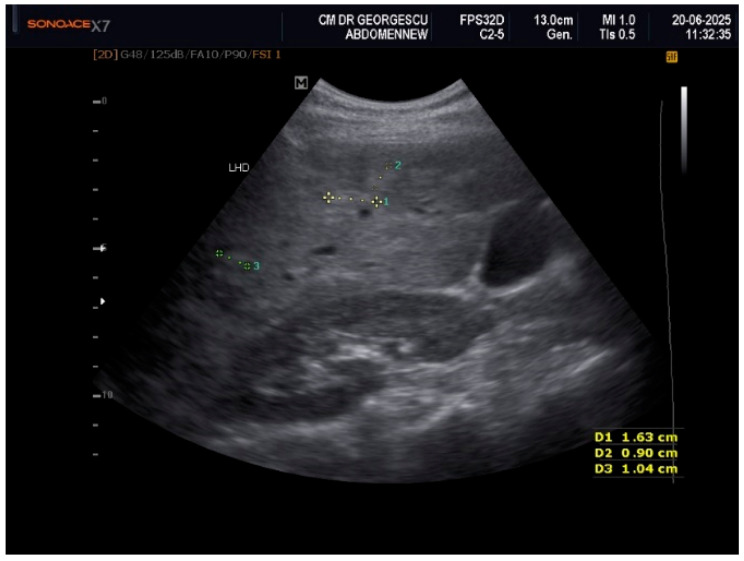
Two-dimensional ultrasound exam features of scattered hyperechoic nodules.

**Figure 3:**
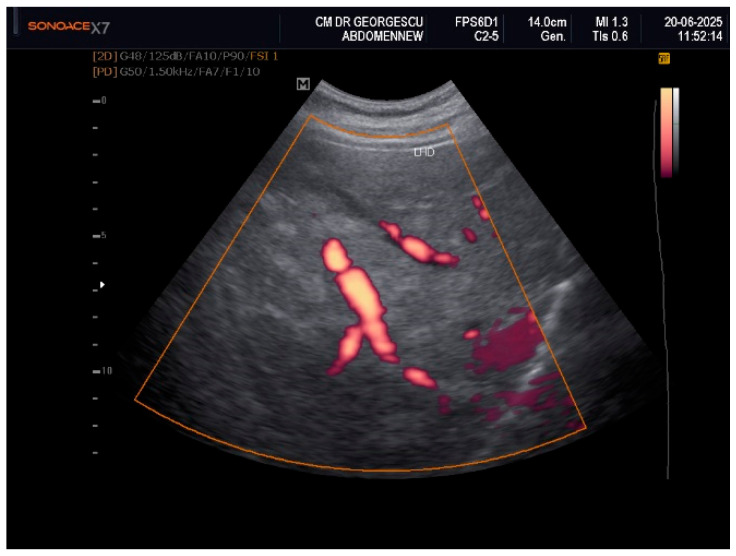
Power Doppler: No mass effect or invasiveness of liver hyperechoic nodules.

Ultrasound examination of the soft superficial formations, performed with a linear probe of 10–12 MHz, revealed aspects of ectopic fat storage, consistent with lipomas. Examination with the convex array 3.5–5 MHz probe permitted visualization of the intra-abdominal structures. The liver appeared with multiple nodular lesions with an echogenic aspect, scattered all over, of various forms and dimensions. The nodules displayed a slightly homogeneous structure with no signs of compression or invasion of the adjacent structures. Doppler examination did not reveal any vascular signal within these formations. These aspects are displayed in Figure 1, Figure 2 and Figure 3. Hemodynamic parameters concerning the hepatic and portal vein, as well as the main hepatic artery, showed a normal range. Liver elastography performed by shear wave method with an Acuson S-3000, high-resolution Ultrasound Equipment (Siemens AG, DE), provided a liver tissue deformation speed of 1.3 m/sec, representing a F0-F1stage of fibrosis. The gallbladder presented with sludge and a calculus of 0.8 cm in diameter. The pancreas presented with a hyperechoic aspect. The abdominal aorta showed mild echogenic plaques.

Even if the clinical appearance suggested a late onset of an acquired partial lipodystrophy, the multi-nodular aspect of the liver, noticed by the initial ultrasound examination, raised many differential diagnosis debates. Various conditions, either malignant, such as liver metastases, or benign, such as multiple hemangiomas, adenomas, nodular focal hyperplasia, infectious/inflammatory lesions, or focal steatosis, should have been ruled out.

Fasting venous blood samples were collected for baseline lab work-ups, as represented in Table 1. Baseline biological data revealed moderate cytolytic syndrome, mild pancreatitis, mixed dyslipidemia, and glucose intolerance. A lot of initial lab work-ups came negative, such as the following: viral screening serology (HB s Ag and anti HCV Ab), tumoral markers (CEA, CA19-9, AFP, CA125, CA15-3, calcitonin), quantiferon TB gold test, autoimmune panels for collagen diseases, autoimmune hepatitis, and celiac disease, as well as PCR test for stool’s parasites and fecal *H. pylori* antigen. However, an autoimmune thyroiditis (Hashimoto’s thyroiditis) was diagnosed, for the time being, with no functional impact.

Given the age and the clinical context, it was mandatory to rule out the possibility of various malignancies, so that further studies were ordered.

Upper and lower digestive endoscopies came out negative for cancer. Antral gastritis, negative for *H. pylori* infection, minor hiatal hernia with secondary gastroesophageal reflux disease, and uncomplicated hemorrhoidal disease were, however, observed.

The gynecological report revealed mild menopausal alterations but came back negative for malignancies.

Cardiac evaluation: Twelve-channel ECG displayed no pathological aspects. ECG Holter examination did not reveal ischemic issues or rhythm disturbances. Transthoracic echocardiography, performed with high-resolution equipment Vivid GE e90 2020 (GE, Health Care, 3000 N Grandview Blvd, Waukesha, WI 53188-1615, USA), described a mild septal hypertrophy, no valvular involvement, no aortic dilatation, left ventricular ejection fraction of 53%, no pulmonary hypertension, and E/A = 0.9.

Contrast-enhanced thorax helical computer tomography (CT) did not show any relevant or suspicious aspects but confirmed that the two superficial tumors, also examined by soft tissue ultrasonography, had a tissue density consistent with lipomas. To appropriately assess the body fat quantity and its distribution, a DXA examination was performed, which showed that the ratio of the fat mass, respectively, the ratio between the percentage of the trunk fat mass and the percentage of the lower body fat mass, was about 1.5. This range was highly suggestive of a partial lipodystrophy with a lower percentage of the upper body fat in a female subject.

**Figure 4:**
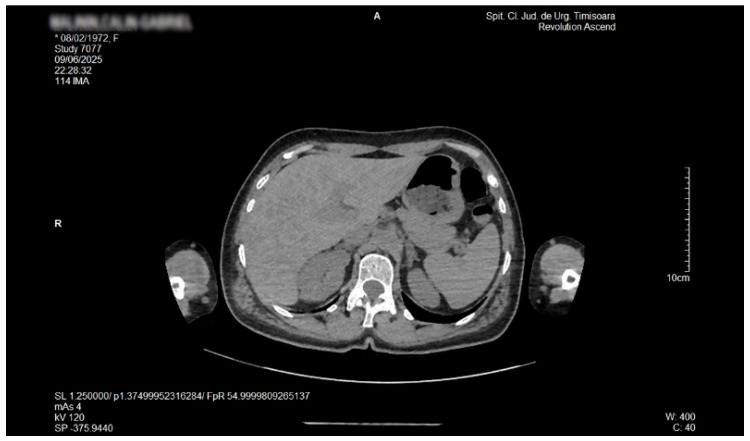
Native axial abdominal CT. Numerous low-attenuation foci scattered throughout the hepatic parenchyma, reflecting a disseminated pattern of lipid infiltration consistent with focal hepatic lipodystrophy.

**Figure 5:**
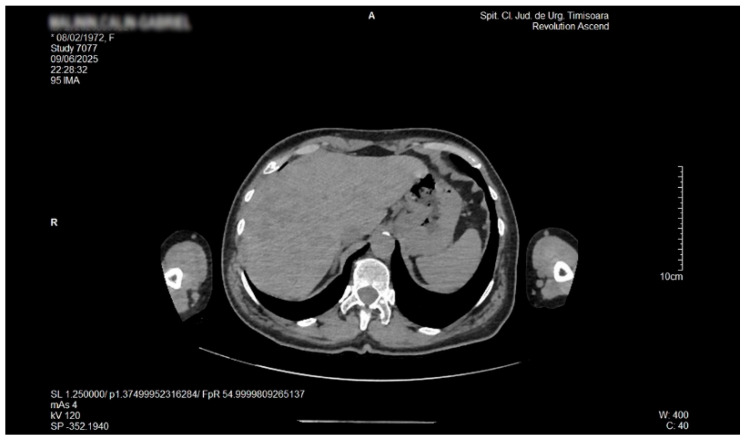
Native axial abdominal CT. Axial non-contrast CT image at a slightly lower hepatic level showing persistent multifocal hypodense lesions, highlighting the diffuse distribution of parenchymal lipid deposition characteristic of the focal disseminated steatosis variant.

Contrast-enhanced abdominal and pelvic helical CT (Siemens Somatom Force dual-source 384-slice scanner, Siemens AG, DE) showed multiple liver hypodense lesions, as illustrated in Figure 4 and Figure 5. The analysis of the hepatic nodular tissue density suggested a multi-nodular fat tissue deposition. A mild edematous pancreas with uniform enhancement was also mentioned.

**Figure 6:**
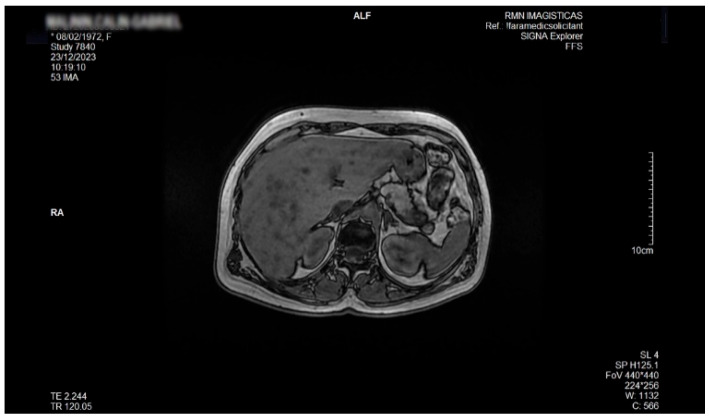
Axial out-of-phase MRI sequence demonstrating multiple disseminated focal hepatic lesions that exhibit marked signal drop, indicating intracellular lipid accumulation consistent with focal hepatic lipodystrophy.

As illustrated in Figure 6, contrast-enhanced magnetic resonance imaging (MRI), Siemens Magnetom Sola 1.5-T system (Siemens Erlangen, DE), showed that nodular liver lesions had similar enhancement to the surrounding liver (isointense lesions), and no mass effect was observed. The out-of-phase MRI sequence highlights intracellular fat by capturing the liver at a moment when signals from fat and water naturally oppose each other, leading to a visible drop in signal in areas containing lipid. Comparison of in-phase and out-of-phase images allowed confident identification of hepatic fat deposition. Venous-phase MRI following intravenous gadolinium administration showed no enhancement within the diffuse focal hepatic lesions, supporting their characterization as non-vascular fat-containing areas, typical of focal hepatic lipodystrophy.

Given the clinical history and the suggestive findings at the physical examination, with characteristic symmetrical near-total loss of subcutaneous fat in the upper part of the body, associated with DXA and abdominal CT results, the diagnosis of partial lipodystrophy seemed highly reasonable. However, further lab work-ups were ordered, such as the HOMA-IR index and leptin range. Results highlighted the insulin resistance and the decreased level of adipokines, consistent with the lipodystrophy diagnosis. To note, blood C3 had normal values.

Taking into account that she denied any family relevant medical history and her fat loss has recently started, not in childhood, no genetic work-ups for inherited lipodystrophy syndromes were considered necessary.

All biological work-ups performed in this patient with liver lipodystrophy, respectively, from blood, urine, and stool samples, are illustrated in Table 1.

Results of the stool’s examination performed by the next-generation sequencing technique are displayed in Table 2.

As illustrated in Table 2, the patient presented with a severe intestinal dysbiosis, enterotype 1 (*Bacteroides* spp. dominant), and multiple alterations of the bioindicators, as well as the bacterial metabolism and the functional gut bacteria.

**Figure 7:**
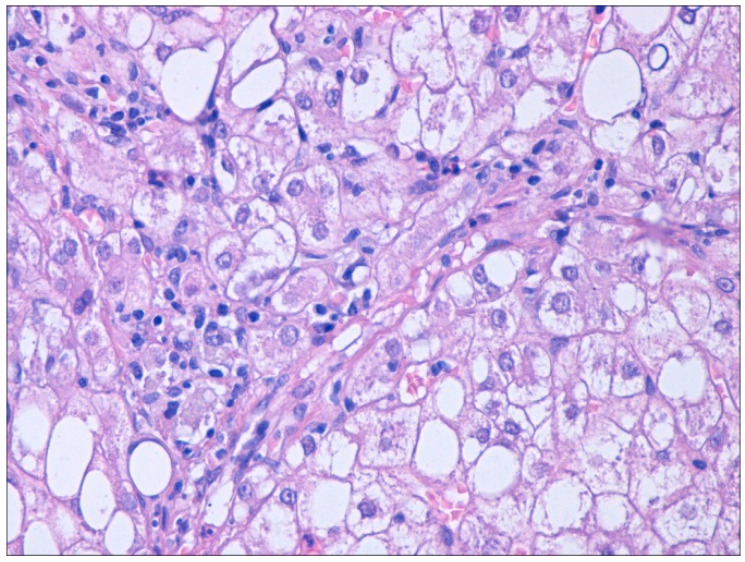
Liver macro-vesicular steatosis, hepatocytes ballooning, and mild inflammatory infiltration of the periportal spaces. HE stain ×400. NAS Score = 6.

**Figure 8:**
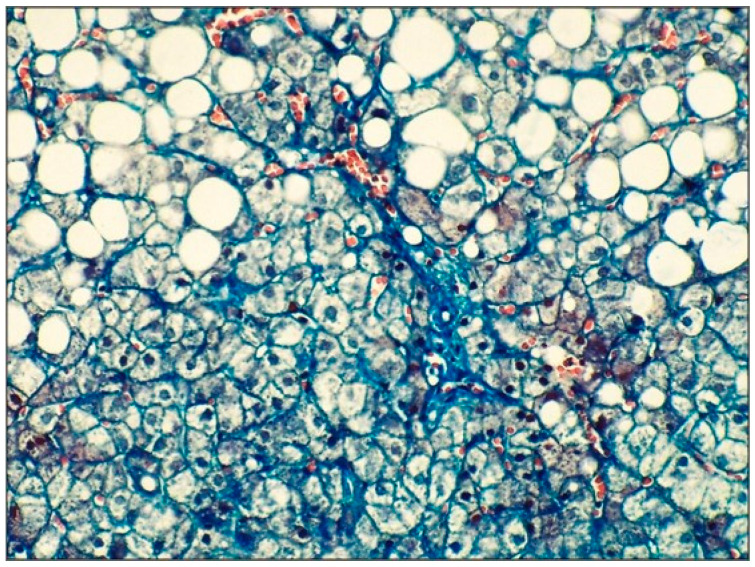
Liver steatosis, mild portal fibrosis, stage 1A. Trichrome Masson stain ×400.

Patient postponed transabdominal ultrasound-guided liver biopsy. A few months later, a liver fragment was, however, obtained by a laparoscopic approach during an emergency cholecystectomy procedure for symptomatic gallstone disease, transported in a saline solution 9‰ to the pathology lab, and further processed. After pre-fixing in buffered 10% neutral formalin, the tissue material was processed by the standard method of paraffin inclusion. From the paraffin blocks, 3 μ sections were made, which were later stained with hematoxylin–eosin (HE) and trichrome Masson method. The slides were scanned with the help of the 3DHISTECH PANNORAMIC MIDI scanner (Öv utca 3-5, H-1141 Budapest, Hungary). Images were taken from the scanned slides at 40× magnification. As presented in Table 3 and Table 4, as well as in Figure 7 and Figure 8, the pathology examination revealed predominantly macro-vesicular steatosis, hepatocyte degeneration with ballooning appearance, mild inflammatory infiltration of the periportal spaces, occasionally foci of necrosis and apoptosis, the total NAS score represented the sum of scores for steatosis, lobular inflammation, and ballooning, ranging from 0 to 8, according to NAS classification [16], consisted of grade 2 (moderate MASH) and stage 1 fibrosis.

In the context of the lipodystrophy diagnosis, a multidisciplinary approach was performed. The patient was advised to go for lifestyle changes, with a low-fat diet and exercise. Statins and fibrates were used to manage high cholesterol and triglycerides. ACE inhibitors and diuretics were continued for blood pressure management. Referrals to an endocrinologist and a diabetologist were made in order to assess when leptin replacement therapy would be beneficial. It was decided to postpone Metreleptin until HbA1c exceeds 7.5%. She was also proposed to take glucagon-like peptide-1 (GLP-1) agonists (Semaglutide), but, given the pancreatitis recent history, the treatment was postponed for the moment. She was given silymarin, however, as hepatoprotective therapy. Given the severity of the gut dysbiosis, the patient received synbiotics and sodium butyrate as supplements.

## 3. Discussions

APL is a rare metabolic disease of uncertain etiology that might frequently be mistaken for other pathologies as a result of its clinical heterogeneity. A female preponderance appears to exist in APL that characterizes BSS. Lipodystrophies, either congenital or acquired, generalized or partial forms, share the same metabolic disturbances characterized by insulin resistance, type 2 diabetes mellitus, dyslipidemia, and a decrease in the leptin range. Many immunity issues are also reported in patients with BSS [17]. The female patient presented in this paper was diagnosed with lipodystrophy as a consequence of a suggestive phenotypic lean aspect, which was associated with liver steatosis, without a history of alcohol abuse or significant exposure to toxins. A characteristic metabolic context with leptin decrease, insulin resistance, and dyslipidemia was also useful in setting the diagnosis.

A large body of the literature is dedicated to diffuse hepatic steatosis in lipodystrophy, whose natural history may mimic the fatty liver condition of the common metabolic syndrome. To note, the hepatocytes’ lipid accumulation may sometimes manifest not only as asymptomatic fat accumulation but also as a severe liver condition, such as MASH, and may progress to advanced fibrosis, cirrhosis, and its complications.

The sequence of liver tissue damages, apart from diffuse fat accumulation, may involve the intervention of oxidative stress and inflammation events, favoring the progression to fibrosis. In this complex pathogenic equation that is yet not precisely defined, insulin resistance represents an important progression cofactor. Interestingly, by the time the end-stage liver disease develops, hepatocytes’ fat accumulation becomes less obvious [18,19,20].

Given the possibility of an advanced liver disease at the time of lipodystrophy diagnosis and the potential of severe outcome, it is mandatory to assess right away the stage of liver disease [21,22].

On the contrary, data related to the natural history of focal, multinodular liver steatosis in lipodystrophy are sparse. Usually, liver steatosis manifests as diffuse hepatic involvement. But in rare cases, the fat deposition may display a spotty, nodular feature, generating many challenges regarding the differential diagnosis. A first case report with lipodystrophy and nodular fatty liver, associated with skeletal modifications, was published in 2002 [23].

To our knowledge, the present case seems to be the second one to report a multiple nodular fatty liver condition, associated with acquired partial lipodystrophy, in a female human subject. Our patient presented with liver lipodystrophy characterized by multifocal liver steatosis that eventually mildly affected the hepatic work-ups. The histology exam showed elements of mild severity of MASH, such as NAS score of 6, with mild hepatocyte ballooning, mild inflammatory infiltrates, and minimal fibrosis with a score of 1A.

It is a clinical reality, supported by prospective studies, that some patients with liver steatosis will never progress to steatohepatitis, fibrosis, or cirrhosis, suggesting that many other cofactors might intervene in the pathophysiology of the MASH or more severe hepatic alterations. One of the factors involved in the progression of the liver lesions might be represented by the gut microbiota dysbiosis. Liver damage related to steatosis may progress as a consequence of the malfunction of the gut-liver axis. The changes noted in the gut–liver axis in patients with steatohepatitis are represented by the alterations of the intestinal microbiota, resulting in dysbiosis with a decrease in microbial biodiversity, small intestinal bacteria overgrowth (SIBO), an increase in lipopolysaccharide-producing bacteria, and an increase in intestinal permeability. All these microbiota changes will trigger the production of proinflammatory cytokines, consecutively activating a damage cascade of the liver, eventually resulting in excessive hepatic lipid storage, lipotoxicity, inflammation, and cellular death. GLP-1 secretion, closely related to short-chain fatty acids (SCFAs), as important byproducts of the gut microbiota, as well as insulin sensitivity, is linked to gut microbiota in patients with MASLD. Leaky gut will promote the translocation of microbial endotoxins and other bacterial byproducts into the bloodstream, with severe consequences for the whole body, including the liver. Hepatocyte mitochondrial alterations in relation to gut dysbiosis were highlighted by many studies in patients with MASLD/MASH. According to recent studies, among many alterations of the gut microbial signature, *Prevotella capri*, a Gram-negative bacterium, seems to play a key role in liver damage by promoting proinflammatory cytokines and non-coding RNAs [24,25].

The female patient presented in this paper displayed intestinal dysbiosis, with a severe decrease in the Shannon index of the alpha biodiversity, many alterations of the bioindicators, of functional bacteria, and bacterial metabolism as well. Highlighting the gut dysbiosis may also provide a customized line of treatment in patients with liver lipodystrophy.

However, it is not only MASLD that is an important burden in patients with lipodystrophy but also the extra-hepatic comorbidities. These pathologies related to metabolic complications, such as cardiovascular and chronic kidney conditions, as well as malignancies, will significantly decrease the life span of patients with various forms of lipodystrophy. Cardio-vascular diseases in lipodystrophy may manifest as hypertrophic cardiomyopathy, left ventricular dysfunction, calcific aortic valve, arrhythmias, and conduction system abnormalities, as well as hypertension. All these entities may at some point progress to atherosclerosis, coronary artery disease, and heart failure, contributing to early mortality in these patients [26,27]. The female patient diagnosed with liver lipodystrophy displayed hypertension and septal left ventricular hypertrophy and had already experienced an episode of a transient cerebral ischemic attack. Autoimmune disorders are common in patients with BSS. Some patients may have an increased susceptibility to infection due to C3 deficiency [28]. As others reported, we have also noted in this female patient some immunity issues, represented by an autoimmune thyroiditis, but no C3 deficiency was observed.

Patients with lipodystrophy have a high risk not only of MASLD/MASH but also of pancreatitis development, which may manifest as a serious complication, especially if the range of hypertriglyceridemia is high or in cases of association with type 2 diabetes mellitus. Dyslipidemia is a major comorbidity and represents an important risk factor for atherosclerosis, and sometimes may manifest with eruptive xanthomas [29,30]. The patient we have presented, developed a mild acute pancreatitis, caused not only by dyslipidemia but also by the associated GSD.

The association of chronic kidney disease (CKD) with MAFLD has already been highlighted by several studies, this particularity being shared by patients with lipodystrophy syndromes, as well. As opposed to patients with metabolic syndrome, those with lipodystrophy might have a higher risk for an early onset of kidney disease, resulting in an augmentation of disease burden and decreasing life span [31,32]. The patient we have discussed here did show, apart from the liver and cardio-vascular issues, a mild decrease in the e-GFR and CKD stage 2. In this context, she should be followed up not only for her liver condition but also for her kidney function, at least once per year.

Leptin replacement therapy in generalized or partial lipodystrophy syndromes, as an adjuvant in patients with persistent high levels of HbA1c and/or triglycerides, in the context of optimized use of hypolipidemic and antidiabetic drugs, showed promising results [33,34].

The rapid worldwide increase in the incidence of obesity and metabolic-associated disorders placed MASLD in a position of global health concern. Although many advances in understanding the metabolic underlying issues are currently reported, very limited therapeutic interventions against MASLD are yet truly available. First steps of therapeutic approaches for MASLD/MASH still rely on lifestyle and dietary modifications, respectively, weight loss, exercise, and dietary shift. There was some evidence, from the last decade of research, that pioglitazone and vitamin E therapy may reduce liver steatosis and inflammation, but no clinical data are yet available related to the long-term results of the use of these drugs in patients with MASH. To date, promising results have been recently shown by clinical trials using Resmetirom, a thyroid liver-specific hormone receptor (THR) beta-selective agonist. Activation of THR-β is linked to important local metabolic improvement. These benefits are related specifically to liver decrease in lipid levels, promotion of bile acid synthesis, fat oxidation, optimization of mitochondrial biogenesis and functionality, also making the extrahepatic side effects of this therapy very limited. That is why the FDA approved in 2024 the use of Resmetirom as the first-line medication for MASH in the USA. Other promising drugs are currently in different stages of clinical trials. Among these therapeutic interventions GLP-1 receptor agonists—Semaglutide or Tirzepatide/Survodutide (GLP-1/GIP coagonis)—may alleviate MASH by decreasing the severity of the fatty liver and improving the inflammation. However, GLP-1 receptor agonists have not shown an important impact on fibrosis regression and were associated with high incidence of digestive side effects. FGF21 analogs—Pegozafermin/Efruxifermin—are other endogenous hormones that regulate lipid and glucose metabolism and may lower the liver lipid storage, alleviate fibrosis, and also improve adipose tissue metabolism. However, frequent digestive side effects have been reported in patients taking this medication.

These novel therapies not only succeeded in reducing the hepatocytes’ fat build-up but also improved the fibrosis range in patients with MASH [35,36]. In terms of specific and efficient therapy against MASH, the patient presented in this case report had very limited therapeutic options. For now Resmetirom was not approved in the European Community, and for the treatment with GLP-1 agonists, she was not a suitable candidate.

Due to the growing evidence of gut microbiota dysbiosis involvement in hepatic steatosis, using probiotics as a therapeutic intervention seems very reasonable. Many recent studies reported that several friendly bacteria, synergistically combined, based mainly on *Lactobacillus* and *Bifidobacterium* spp., have been successfully used in treating patients with MASLD/MASH [37]. In the context of MASH-associated severe gut dysbiosis, with multiple bacterial alterations, we decided to recommend the patient synbiotics and sodium butyrate as supplements.

An important therapeutic target in patients with lipodystrophy remains the addressing of the lipid and carbohydrate metabolic disorders. Depending on the severity of dyslipidemia, several therapeutic interventions may be used, such as statins, ezetimide, and alirocumab, to achieve the LDL-cholesterol targets. In cases of severe hypertriglyceridemia, fibrates and omega-3 fatty acids may be required. Glucose intolerance or type 2 diabetes mellitus should also be carefully addressed. GLP-1 agonists should be the first to consider, given the promising results in treating the MASH condition [38,39].

## 4. Conclusions

Given their heterogeneity and rarity, lipodystrophy syndromes may be either overlooked or misdiagnosed as other entities. BSS may be associated with liver disease, including cirrhosis and liver failure. Liver lipodystrophy in BSS may sometimes feature steatosis with a focal, multi-nodular aspect, multiplying the diagnostic burden. Liver lipodystrophy may manifest as asymptomatic fat accumulation but may progress to severe conditions, representing one of the major causes of mortality in BSS, apart from the cardio-vascular comorbidities. Given the potential of severe outcomes, it is mandatory to correctly assess the stage of liver disease since the first diagnosis.

## Figures and Tables

**Table 1:** Baseline lab work-up data.

Variables	Reference Ranges	Actual Range	Variables	Reference Ranges	Actual Range
Hb g/L	12–15	13.6	LDL-Chol mg%	<100	143
Leu/mm3	4000–10,000	6770	HDL-Chol mg%	>50	42
Plt/mm3	150,000–450,000	230,000	TRG mg%	<150	356
ALAT U/L	7–40	65	FBS mg%	<100	112
ASAT U/L	8–35	32	HbA1c	<5.7	6.3
ALP U/L	40–129	41	HOMA-IR	<1	3.2
GGT U/L	5–40	57	Leptin ng/mL	0.5–15.2	0.3
TBil mg/dL	0.1–1.2	0.8	TSH UI/mL	0.4–4.5	4.7
Lipase U/L	2–160	723	FT3 pg/mL	2.3–4.2	3.5
FIB4	<1.3	0.91	FT4 pg/mL	0.8–1.9	0.9
Creat mg/dL/eGFR mL/min/1.73 m^2^	0.6–1.1	0.94/78	anti-TPO Ab IU/mL	<35	98
UACR mg/g	<30	100	Anti-thyroglobulin Ab IU/mL	<115	176
CRP g/dL	0.3–0.5	1.2	C3 mg/dL	80–200	154

Hb = hemoglobin; Leu = leukocytes; Plt = platelets; ALAT = alanine-aminotransferase; ASAT = aspartate-aminotransferases; ALP = alkaline phosphatase; GGT = gamma-glutamyl-transpeptidase; TBil = total bilirubin; creat = creatinine; FIB4 = Fibrosis 4; UACR = urinary albumin creatinine ratio; CRP; = C-reactive protein; LDL-Chol = low-density lipoprotein-cholesterol; HDL-Chol = high-density lipoprotein-cholesterol; TRG = triglycerides; FBS = fasting blood sugar; HbA1c = glycosylated hemoglobin; HOMA-IR = Homeostasis Model Assessment Insulin Resistivity; TSH = thyroid stimulating hormone; FT3 = free triiodthyronine; FT4 = free thyroxine; TPO = thyroid peroxidase; C3 = complement 3 fraction.

**Table 2:** Stool’s microbiota report.

Gut Microbiota Alterations		Range
Shannon index		2.39
*F/B* ratio		0.8
Gut dysbiosis severity		3
LPS + bacteria	*Citrobacter* spp.	0.02
	*Providencia* spp.	0.002
	*Seratia* spp.	0.018
	*Suterella* spp.	4.001
Mucin-degrading bacteria	*Akkermansia muciniphila*	0.000
	*Prevotella* spp.	0.000
	*Prevotella capri*	0.001
Mucosa-protective bacteria	*Akkermansia muciniphila*	0.000
	*Faecalibacterium prausnitzii*	0.008
Neuroactive bacteria	*Bifidobacterium adolescentis*	0.625
	*Lactobacillus brevis*	0.000
	*Lactobacillus paracasei*	0.000
	*Alistipes* spp.	0.021
Butyrate-producing microbiota	*Eubacterium* spp.	0.011
	*Faecalibacterium prausnitzii*	0.008
	*Roseburia* spp.	0.000
	*Ruminococcus* spp.	6.850
Enterotype		1

*F/B* = *Firmicutes/Bacteroidetes*, LPS = lipopolysaccharides.

**Table 3:** MASH grading.

Variables	Score
Steatosis	2
Ballooning obvious, predominantly zone 3	2
Inflammation, polymorphs, and chronic inflammation	2
Portal inflammation	Mild to moderate

**Table 4:** MASH staging.

Stage 1	Zone 3 perisinusoidal/pericellular fibrosis, focal or extensive	score 1A

## Data Availability

The original contributions presented in this study are included in the article. Further inquiries can be directed to the corresponding author.

## Abbreviations

The following abbreviations are used in this manuscript:

BSS	Barraquer–Simons syndrome
APL	Acquired partial lipodystrophy
MASLD	Metabolic dysfunction-associated steatotic liver disease
MASH	Metabolic disease-associated steatohepatitis
FIB-4	Fibrosis 4
MRI	Magnetic resonance imaging
BMI	Body Mass Index
BP	Blood pressure
MHz	Megahertz
TB	Tuberculosis
HB s Ag	Hepatitis B surface antigen
anti-HCV Ab	Anti-hepatitis C virus antibodies
CEA	Carcino-embryonic antigen
CA19-9	Cancer antigen 19-9
AFP	Alpha fetoprotein
CA125	Cancer antigen 125
CA15-3	Cancer antigen15-3
PCR	Polymerase chain reaction
ECG	Electrocardiogram
CT	Computed tomography
DXA	Dual energy x-ray absorptiometry
HOMA-IR	Homeostasis Model Assessment Insulin Resistivity
LPS	Lipopolysaccharide
HE	Hematoxylin–eosin
HbA1c	Glycosylated hemoglobin
GLP-1	Glucagon-like peptide-1
SIBO	Small intestinal bacteria overgrowth
SCFAs	Short-chain fatty acids
RNA	Ribonucleic acid
C3	Complement fraction C3
CKD	Chronic kidney disease
eGFR	estimated-glomerular filtration rate
THR	Thyroid hormone receptor
